# Electromyographic analysis of muscle activation and co-activation across smash phases in para-badminton: an exploratory investigation of neuromuscular coordination

**DOI:** 10.3389/fbioe.2026.1853282

**Published:** 2026-08-31

**Authors:** Khaled Abuwarda, Abdulazeem Alotaibi, Abdel-Rahman Akl

**Affiliations:** 1 Department of Physical Education and Kinesiology, College of Education, Qassim University, Qassim, Saudi Arabia; 2 Faculty of Sports Science-Abo Qir, Alexandria University, Alexandria, Egypt

**Keywords:** co-activation, injury prevention, physical activity, sports exercise, wearable sensors

## Abstract

**Objective:**

This study aimed to examine and compare muscle activation and co-activation across the backswing and forward swing phases of the para-badminton smash to improve understanding of phase-specific neuromuscular coordination that may inform future biomechanical and injury-related research.

**Methods:**

Nine elite para-badminton players were recruited for the study (body mass: 79.0 ± 7.9 kg; height: 172.1 ± 5.2 cm). Surface electromyography (sEMG) signals were recorded using a wireless EMG system, and data were collected from eight muscles (flexor carpi ulnaris, extensor carpi ulnaris, biceps brachii, triceps brachii, infraspinatus, latissimus dorsi, rectus abdominis, and erector spinae longissimus).

**Results:**

The results indicated that the wrist (flexor carpi ulnaris and extensor carpi ulnaris) and elbow (biceps and triceps) muscles showed significantly greater activation during the forward swing (*p* < 0.01–0.001), with peak activity in the early-to-mid acceleration phase; however, only the wrist demonstrated a significant change in the co-activation index (CoI) (*p* < 0.001), while elbow agonist–antagonist balance remained stable. At the shoulder, the infraspinatus maintained consistent activation, whereas the latissimus dorsi was selectively recruited during the forward swing (*p* < 0.01), resulting in an increased CoI (*p* < 0.001). Similarly, trunk muscles exhibited phase-specific behavior, with stable erector spinae activity and significant rectus abdominis recruitment during the forward swing (*p* < 0.001), leading to elevated trunk CoI. Overall, these findings indicate coordinated, segment-specific modulation of activation and co-activation to support force generation and stabilization during the acceleration phase.

**Conclusion:**

Overall, the findings indicate that muscle co-activation is segment-specific and adapted to the mechanical demands of each joint within the kinetic chain. Imbalances such as excessive wrist co-activation or insufficient proximal stabilization may increase injury risk. Therefore, injury prevention in para-badminton should focus on optimizing wrist stability, maintaining elbow muscle balance, enhancing shoulder control, and improving trunk stability to support efficient force transfer and performance.

## 1 Introduction

Badminton remains one of the most widely practiced sports globally, owing to its accessibility across diverse age groups and genders (Kusnaedi et al., 2025). In parallel, para-badminton has developed into a dynamic and inclusive discipline, providing athletes with physical impairments the opportunity to compete at elite levels (Tweedy and Vanlandewijck, 2011). As an adapted variant of the sport, para-badminton is specifically structured to accommodate individuals with disabilities, enabling participation based on the nature and extent of their impairments (Strapasson et al., 2019).

A detailed understanding of the disability profiles within the para-badminton population is essential for coaches to design targeted training strategies that enhance performance while minimizing injury risk. Such insights support informed decision-making and contribute to the development of training environments that enable athletes with diverse functional capacities to perform at their highest potential (Blauwet and Willick, 2012). Furthermore, recognizing the effects of different impairments on sports participation and performance is critical for optimizing motor capabilities and overall athletic performance in this population (Sharma et al., 2025).

Wheelchair badminton presents distinct characteristics, particularly in its classification system. Athletes are categorized into two functional classes: wheelchair 1 (WH1) and wheelchair 2 (WH2). The WH1 class includes players with impairments affecting the abdominal muscles and lower limbs, resulting in limited trunk control, whereas the WH2 class comprises athletes with better trunk function but lower limb impairment, often with partial sensory preservation (Alberca et al., 2024).

In general, badminton is a high-intensity sport that requires players to perform multiple movements in a short time at high speed (Masu and Nagai, 2016). It is also classified as an explosive sport (Gusliandi et al., 2020).

During single-handed overhead strokes, the elbow musculature plays a crucial role in controlling elbow joint positioning and coordinating force transmission throughout the movement. Concurrently, the wrist muscles maintain wrist extension and radial alignment while resisting the substantial wrist-extension forces generated during stroke execution (Ju et al., 2021a). Proximally, the shoulder joint is one of the most mobile and functionally versatile joints in the human body due to its ball-and-socket anatomical structure. Consequently, the surrounding musculature is essential for maintaining glenohumeral stability, protecting joint integrity, and preventing muscular imbalances during high-velocity upper-limb actions (Roetert et al., 2024).

Understanding muscle activation patterns during competitive performance is essential for optimizing training strategies and improving movement control. In disability sports, advances in biomechanical research and wearable technologies have become increasingly important for identifying how specific impairments influence sport performance and movement mechanics. Among these technologies, surface electromyography (sEMG) is widely used to assess muscle activation and co-activation characteristics during functional and sport-specific movements, providing valuable insight into neuromuscular coordination and control (Lee et al., 2017).

Therefore, control and stability of movement are important for injury prevention and performance enhancement to maximize the effectiveness of the player’s abilities. In this context, the key factor in stroke control and injury prevention is muscle co-activation, which involves the simultaneous contraction of agonist and antagonist muscles across a joint.

Antagonist muscle co-activation is a physiological phenomenon characterized by the simultaneous activation of muscles opposing the primary agonist action during movement execution. This neuromuscular strategy may help identify alterations in movement mechanics and motor control patterns (Ju et al., 2021b). Furthermore, antagonist co-activation has been proposed as a mechanism for increasing joint stiffness, thereby enhancing multi-segment stabilization, dynamic joint control, and overall kinetic coordination during functional movements (Pincivero et al., 2019).

This co-activation enhances joint stability and motor control, which is especially important during a high-intensity task (Hortobagyi et al., 2009; Nagai et al., 2011). For wheelchair players, proper co-activation is vital to maintaining joint stiffness and controlling the complex movements required during the stroke phases (Garcia-Vicencio et al., 2024). Understanding these muscle activation patterns and their implications on performance and injury risk can provide valuable insights for improving training and rehabilitation strategies.

Despite growing participation and competitive development of para-badminton, scientific evidence regarding the neuromuscular characteristics of wheelchair para-badminton athletes remains limited (Strapasson et al., 2019). Although surface EMG and muscle co-activation analysis are well established in sports biomechanics, their application to wheelchair para-badminton, particularly during high-velocity overhead actions such as the smash stroke, has not been sufficiently investigated. In particular, little is known about the phase-specific coordination strategies of the wrist, elbow, shoulder, and trunk musculature in WH2 athletes, whose movement mechanics are uniquely influenced by impaired lower-limb contribution and increased upper-body demands. Therefore, the purpose of this study was to examine and compare muscle activation and muscle co-activation across the backswing and forward swing phases of the para-badminton smash to improve understanding of phase-specific neuromuscular coordination that may inform future biomechanical and injury-related research.

## 2 Materials and methods

### 2.1 Subjects and study design

Nine elite para-badminton players (WH2) were recruited (body mass: 79.0 ± 7.9 kg; height: 172.1 ± 5.2 cm). An *a priori* power analysis (G*Power v3.1.9.7), based on an effect size (dz = 0.8), α = 0.05, and power = 0.80, indicated a required sample size of 12 participants. The final sample (n = 9) reflects the limited availability of elite para-badminton athletes, a highly specialized population with inherent recruitment constraints. Consistent with research in elite and para-sport settings, the study is, therefore, positioned as an exploratory, hypothesis-generating investigation aimed at providing further insight into muscle activation and co-activation across smash phases in para-badminton. Participants were excluded if they had experienced significant interruptions in their training history (i.e., longer than 6 months) or if they were currently injured or recovering from injuries that could affect performance. All participants provided written informed consent prior to participation. The study protocol was approved by the ethics committee of the hosting institution.

### 2.2 Protocol and data collection

Participants began with a warm-up consisting of 5 minutes of submaximal badminton strokes. Following the warm-up, each participant performed three trials of a smash (100 km/h). The sequence of exercises during the warm-up and experimental trials was kept consistent to ensure standardization. To determine limb dominance, participants were asked which limb they preferred to use during the exercises (Kotsifaki et al., 2022). Smash phases were determined through video analysis using the Simi Motion Capture System (Simi Reality Motion Analysis, Version 9.0.6), synchronized with the EMG system at a sampling rate of 100 frames per second. The smash was divided into two distinct phases: backswing and forward swing. The movement phases were identified using synchronized high-speed video recordings. The backswing phase was defined as the period from the initiation of racket movement after the shuttlecock was released by the ball machine to the instant of movement reversal. The forward swing phase was defined from the end of the backswing (movement reversal) to shuttlecock release. Each participant performed three valid smash trials while the shuttlecock was launched by a ball machine at a standardized speed of 100 km/h, ensuring consistent delivery across all participants. A one-minute rest period was provided between trials to prevent fatigue. The three trials per participant were used to investigate muscle activity and calculate the muscle co-activation index (CoI) (total trials = 27).

### 2.3 sEMG signal acquisition and processing

**FIGURE 1:**
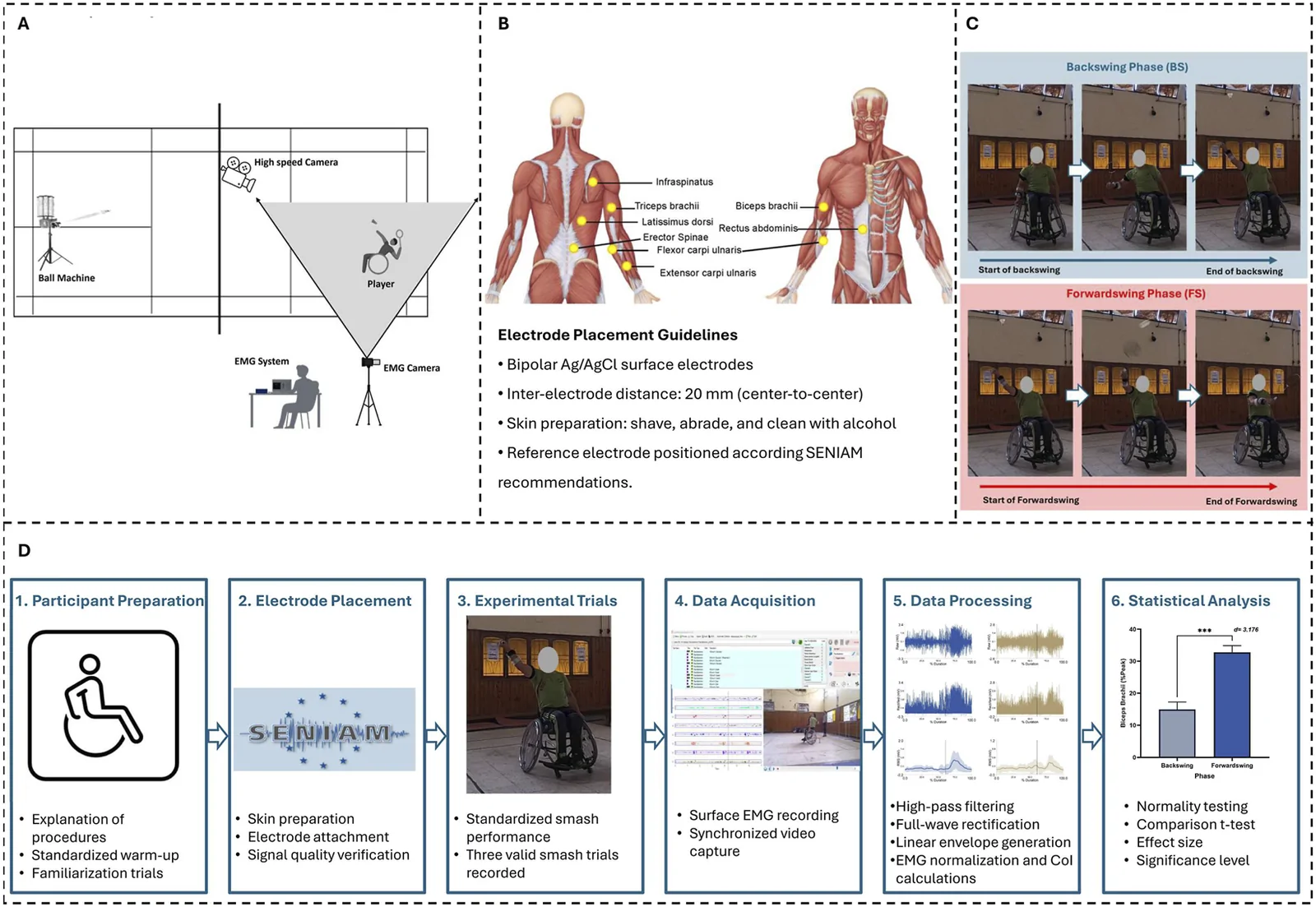
Graphical representation of the experimental setup and procedures for sEMG analysis of the para-badminton smash. **(A)** Experimental setup showing the wheelchair athlete performing the smash task. **(B)** Surface EMG electrode placement on the selected upper-limb and trunk muscles. **(C)** Backswing (BS) and forward swing (FS) phases analyzed during the smash stroke. **(D)** Flowchart summarizing participant preparation, data collection, signal processing, and statistical analysis procedures.

Following SENIAM guidelines, participants’ skin was shaved, lightly abraded, and cleansed with alcohol prior to electrode placement (Hermens et al., 2000). Gel-coated, self-adhesive bipolar surface electrodes (10 mm diameter, silver/silver chloride; SKINTACT FS-RG1/10, Leonhard Lang GmbH, Innsbruck, Austria) were placed with a 2-cm center-to-center distance. Electrodes were positioned on both the dominant and non-dominant sides according to SENIAM recommendations (www.seniam.org) over the following muscles: flexor carpi ulnaris, extensor carpi ulnaris, biceps brachii, triceps brachii, infraspinatus, latissimus dorsi, rectus abdominis, and erector spinae longissimus. sEMG signals were collected using a wireless EMG system (Myon m320RX; Myon, Switzerland) at a sampling frequency of 1,000 Hz and with a 16-bit A/D resolution. Signal processing was performed using Visual3D software (C-Motion, Germantown, MD, United States). The raw EMG signals were processed and analyzed to obtain both temporal activation profiles and normalized quantitative measures. After signal acquisition, the EMG data were high-pass-filtered using a high-pass Butterworth filter with a cutoff frequency of 25 Hz to minimize movement artifacts in the raw EMG recordings. The filtered signals were subsequently full-wave-rectified and low-pass-filtered at 15 Hz to generate the linear envelope of the EMG signal (Quittmann et al., 2020; Akl et al., 2023). The waveform figures, presented in millivolts (mV), represent the averaged linear envelope profiles across the movement cycle and were used to illustrate the temporal characteristics of muscle activation during the backswing and forward swing phases. For statistical analysis, EMG amplitudes were normalized to the peak dynamic activation recorded during the smash trials (%Peak). Dynamic normalization was selected because the study aimed to compare phase-specific muscle activation during a sport-specific task. This approach is particularly appropriate for elite WH2 para-badminton athletes, in whom impairment-related functional limitations and variability in maximal voluntary effort may compromise the reliability of MVIC-based normalization. Consequently, %Peak normalization provided a practical and functional reference for evaluating muscle activation during the para-badminton smash (Hakariya et al., 2026). The normalized EMG amplitudes and CoI values were subsequently used for phase comparisons and statistical testing (Figure 1).

### 2.4 Quantification of the co-activation index

The selected muscle pairs were defined as functional co-activation pairs based on their roles in dynamic joint stabilization and force transmission during the para-badminton smash, rather than as fixed anatomical agonist–antagonist pairs throughout the entire movement. At the elbow, the biceps brachii and triceps brachii were selected as the primary flexor–extensor muscle pair. At the wrist, the flexor carpi ulnaris and extensor carpi ulnaris were chosen because they function antagonistically to regulate wrist stability during racket acceleration and shuttlecock impact. For the shoulder, co-activation was calculated between the latissimus dorsi and infraspinatus, which perform complementary functional roles in shoulder movement and glenohumeral stabilization during the smash. The latissimus dorsi primarily contributes to shoulder extension, adduction, and internal rotation during the acceleration phase, whereas the infraspinatus contributes to external rotation, horizontal abduction, and dynamic stabilization of the glenohumeral joint, particularly during deceleration. Trunk co-activation was determined using the rectus abdominis and erector spinae, representing the principal anterior and posterior trunk stabilizers responsible for trunk stiffness, postural control, and efficient force transmission between the trunk and upper limb during seated overhead movements. Muscle co-activation around the dominant upper-limb joints and trunk was quantified using the CoI (Equation 1).

$$CoI=\frac{\int_{t1}^{t2}{NEMG}_{antagonist}\left(t\right)dt}{\int_{t1}^{t2}\left[{NEMG}_{agonist}+{NEMG}_{antagonist}\right]\left(t\right)dt}\times 100,$$ (1)

where t_1_ and t_2_ represent the beginning and end of each phase, NEMG_antagonist_ denotes the activity of the antagonist muscle, and NEMG_agonist_ denotes the activity of the agonist muscle during the backswing and forward swing phases (Kellis et al., 2003; Oliveira et al., 2017; Akl et al., 2021; Waanders et al., 2021).

### 2.5 Statistical analysis

Descriptive statistics are reported as the means and 95% confidence intervals (mean ± CI). Data normality was assessed using the Shapiro–Wilk test, confirming their suitability for parametric analysis. A paired T-test was used to detect significant differences and compare the mean of each variable during the two phases: backswing and forward swing. Cohen’s d was used to determine the effect size, where values of 0.2, 0.5, and 0.8 represent small, medium, and large differences, respectively (Cohen, 1988). All statistical analyses were conducted using IBM SPSS Statistics version 27 (IBM Corp., Armonk, NY, United States).

## 3 Results

**FIGURE 2:**
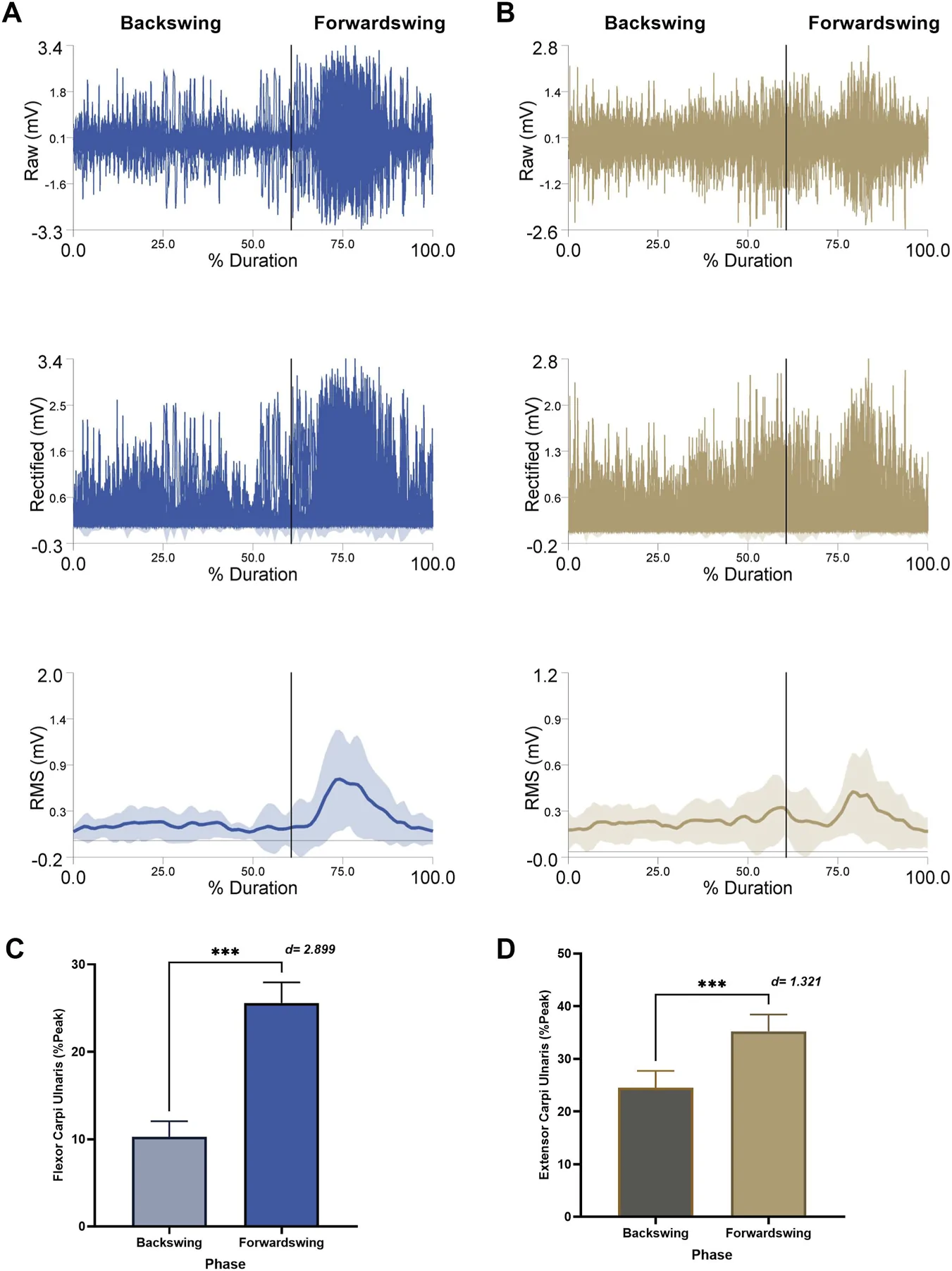
Averaged electromyography (EMG) activity waveforms of the wrist muscles during the para-badminton smash stroke across the backswing and forward swing phases, representing mean data across all participants. Flexor carpi ulnaris **(A)** (first row) raw EMG signal, (second row) rectified EMG, and (third row) linear envelope of the EMG signal. Extensor carpi ulnaris **(B)** (first row) raw EMG signal, (second row) rectified EMG, and (third row) linear envelope of the EMG signal. Mean values and confidence intervals of normalized EMG amplitude (%Peak) are presented for the flexor carpi ulnaris **(C)** and extensor carpi ulnaris **(D)**. Asterisks indicate significant differences between phases (*** *p* < 0.001).

Electromyographic (EMG) analysis of the flexor carpi ulnaris and extensor carpi ulnaris revealed distinct, phase-dependent activation profiles during the smash stroke (Figure 2). In the flexor carpi ulnaris (Figure 2A), raw and rectified signals showed moderate activity during the backswing phase (≈0–60% of the movement cycle). This was followed by a sharp increase in signal amplitude during the forward swing phase (≈60–100%). The linear envelope of the EMG signal further illustrated this modulation, with activation gradually increasing toward the transition point and reaching peak values during the early- to mid-forward swing. A similar trend was observed in the extensor carpi ulnaris (Figure 2B). Signals indicated low activity during the backswing, followed by a marked increase immediately after the phase transition. Large-amplitude bursts occurred throughout the forward swing, with the linear envelope of the EMG signal curve peaking during the mid-forward swing period. For the wrist musculature (Figure 2), both the flexor carpi ulnaris and extensor carpi ulnaris exhibited significantly greater mean activation during the forward swing phase compared to those during the backswing phase (*p* < 0.001; d = 2.899 and *p* < 0.001; d = 1.321, respectively) (Figures 2C,D). Although both muscles showed moderate to low activity during the backswing (0%–60% duration), a sharp increase in signal amplitude and large-amplitude bursts occurred immediately post-transition.

**FIGURE 3:**
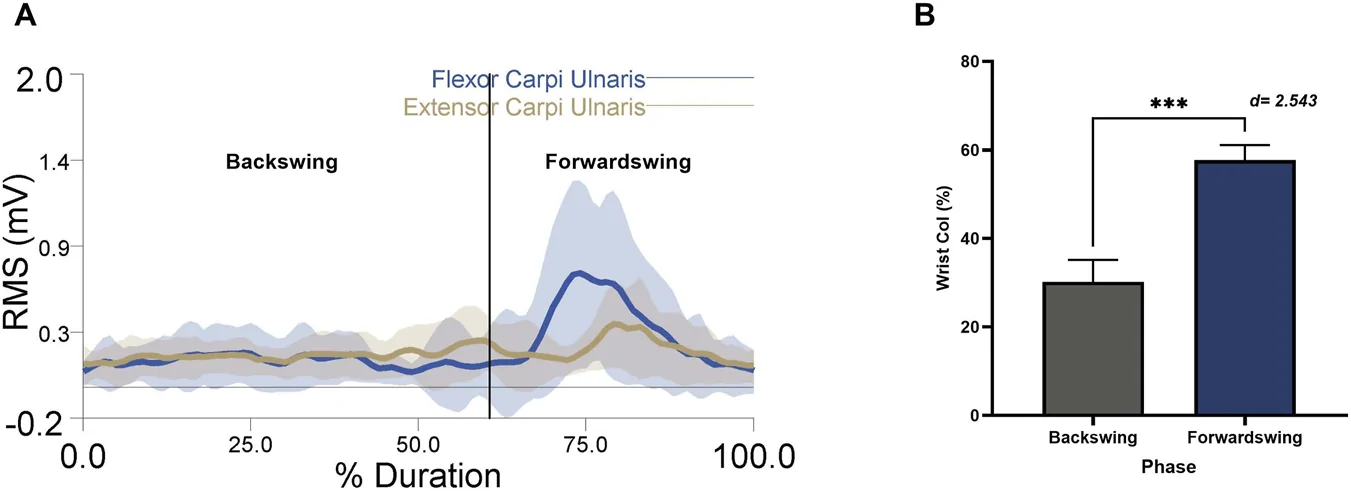
Averaged EMG linear envelope signals of the wrist muscles (flexor carpi ulnaris and extensor carpi ulnaris) during the para-badminton smash stroke across the backswing and forward swing phases. **(A)** Linear envelope of the EMG signal waveforms represented by solid lines, with shaded areas indicating standard deviation across all participants. **(B)** Mean wrist co-activation index (CoI, %) values with 95% confidence intervals during the backswing and forward swing phases across all participants. Asterisks indicate significant differences between phases (*** *p* < 0.001).

Consequently, Figure 3A shows the linear envelope of the EMG signals recorded from the wrist muscles (flexor carpi ulnaris and extensor carpi ulnaris). In addition, the wrist CoI shifted significantly between phases (*p* < 0.001; d = 2.543) (Figure 3B), indicating that the relative balance of flexor–extensor recruitment is dynamically modulated to facilitate high-velocity impact.

**FIGURE 4:**
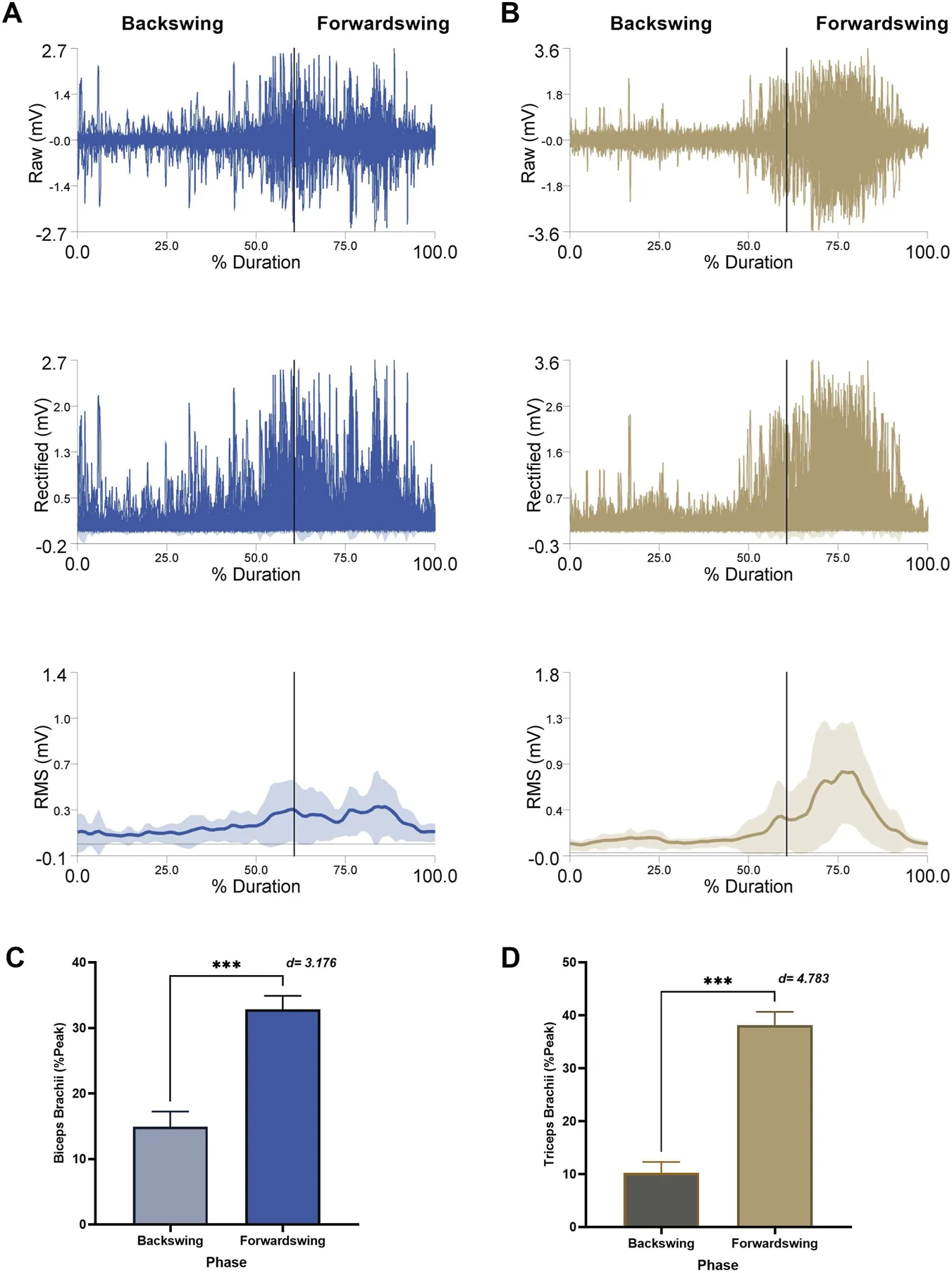
Averaged EMG activity waveforms of the elbow muscles during the para-badminton smash stroke across the backswing and forward swing phases, representing mean data across all participants. Biceps brachii **(A)** (first row) raw EMG signal, (second row) rectified EMG, and (third row) linear envelope of the EMG signal. Triceps brachii **(B)** (first row) raw EMG signal, (second row) rectified EMG, and (third row) linear envelope of the EMG signal. Mean values and confidence intervals of normalized EMG amplitude (%Peak) are presented for the biceps brachii **(C)** and triceps brachii **(D)**. Asterisks indicate significant differences between phases (*** *p* < 0.001).

EMG analysis of the biceps brachii and triceps brachii revealed distinct, phase-dependent activation profiles during the smash stroke (Figure 4). For the biceps brachii (Figure 4A), raw and rectified signals showed relatively moderate activity during the backswing phase (≈0%–60% of the movement cycle), followed by a marked increase in signal amplitude during the forward swing phase (≈60%–100%). The linear envelope of the EMG signals further illustrated this modulation, with activation gradually increasing toward the phase transition and peaking during the early- to mid-forward swing. A similar trend was observed in the triceps brachii (Figure 4B). Signals indicated low baseline activity during the backswing, followed by a sharp increase immediately after the transition point. Large-amplitude bursts were sustained throughout the forward swing phase, with the linear envelope of the EMG signal curve reaching its maximum during the mid-forward swing period. In the elbow (Figure 4), a similar trend was observed for individual muscle recruitment. Both the biceps brachii and triceps brachii demonstrated significantly greater activation during the forward swing (*p* < 0.001; d = 3.176 and 4.783, respectively) (Figures 4C,D).

**FIGURE 5:**
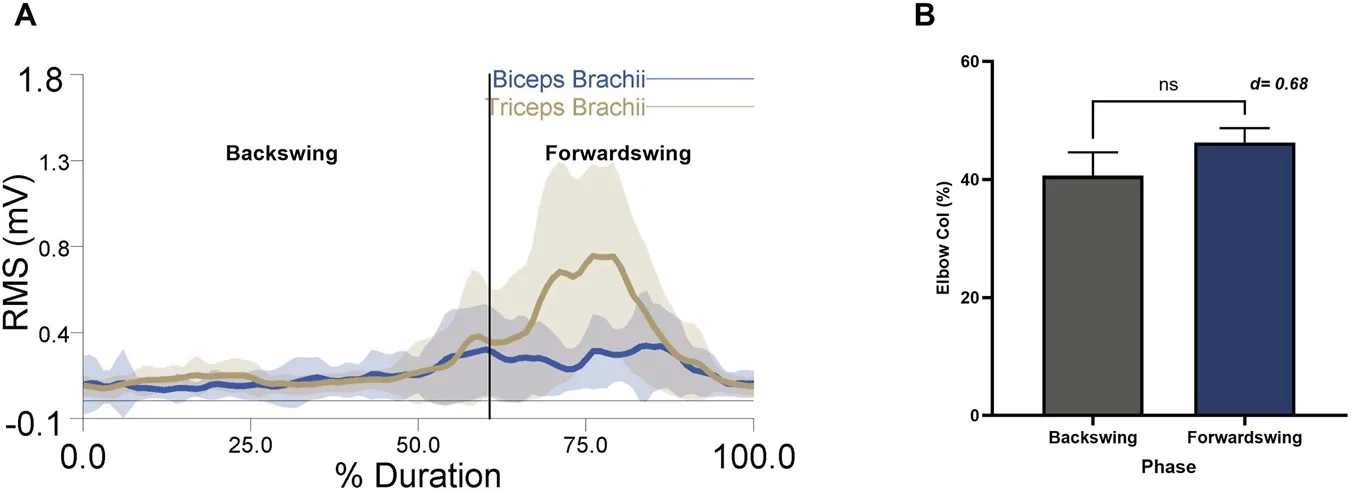
Averaged EMG linear envelope signals of the elbow muscles (biceps brachii and triceps brachii) during the para-badminton smash stroke across the backswing and forward swing phases. **(A)** Linear envelope of the EMG signal waveforms represented by solid lines, with shaded areas indicating standard deviation across all participants. **(B)** Mean elbow co-activation index (CoI, %) values with 95% confidence intervals during the backswing and forward swing phases across all participants. Asterisks indicate significant differences between phases; ns indicates non-significant differences.

Consequently, Figure 5A shows the linear envelope of the EMG signals recorded from the elbow muscles (biceps brachii and triceps brachii). However, unlike the wrist, the elbow CoI did not differ significantly between the backswing and forward swing phases (*p* > 0.05; d = 0.68) (Figure 5B). This suggests that although absolute muscle recruitment increases to drive the stroke, the proportional agonist–antagonist relationship remains stable to provide consistent joint stabilization throughout the movement.

**FIGURE 6:**
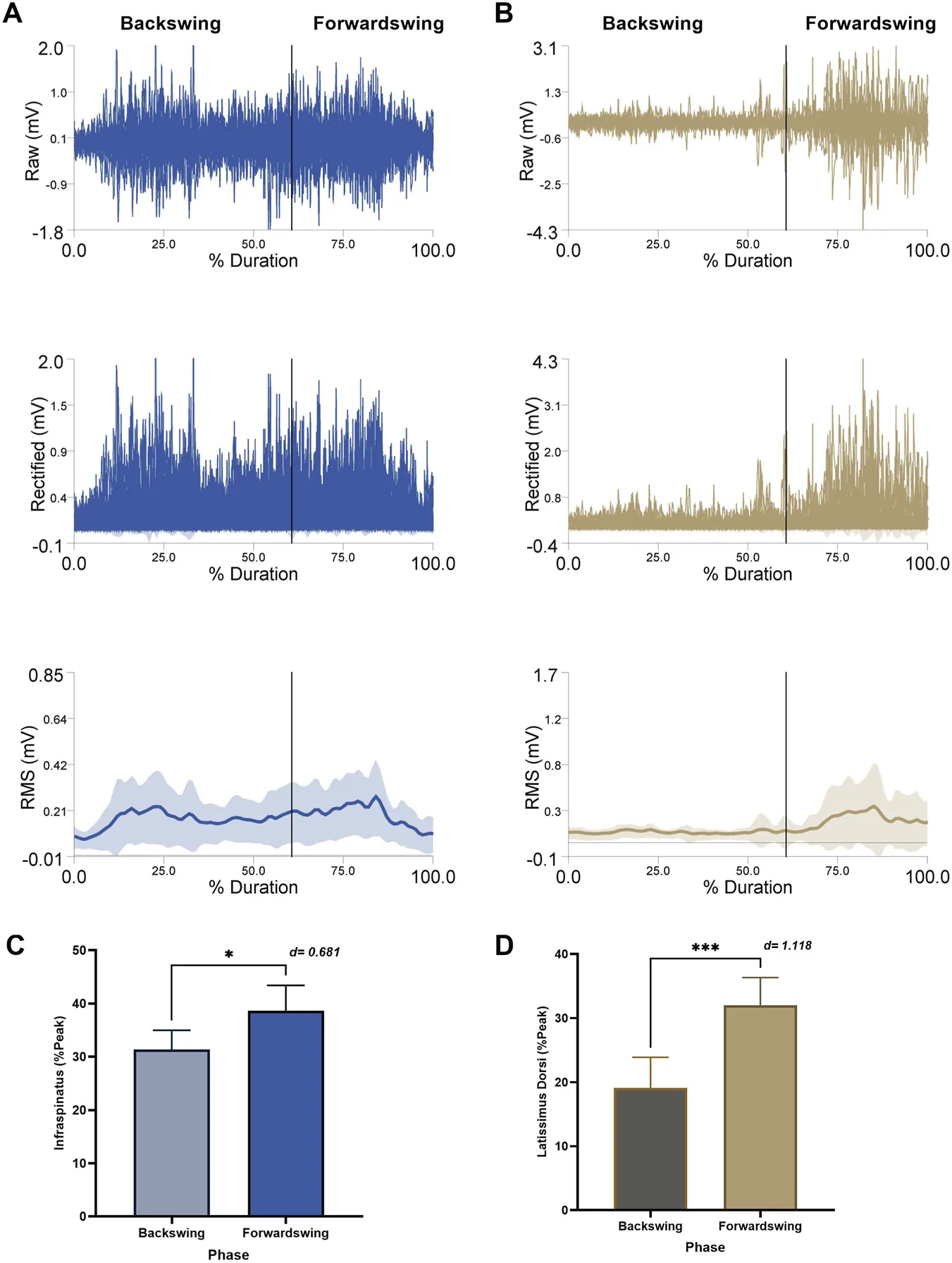
Averaged EMG activity waveforms of the upper trunk muscles during the para-badminton smash stroke across the backswing and forward swing phases, representing mean data across all participants. Infraspinatus **(A)** (first row) raw EMG signal, (second row) rectified EMG, and (third row) linear envelope of the EMG signal. Latissimus dorsi **(B)** (first row) raw EMG signal, (second row) rectified EMG, and (third row) linear envelope of the EMG signal. Mean values and confidence intervals of normalized EMG amplitude (%Peak) are presented for the infraspinatus **(C)** and latissimus dorsi **(D)**. Asterisks indicate significant differences between phases (* *p* < 0.05; *** *p* < 0.001).

The EMG profiles for the infraspinatus and latissimus dorsi during the smash stroke are presented in Figure 6. The infraspinatus (Figure 6A) exhibited relatively stable activation throughout the movement. Both raw and rectified signals showed consistent activity during the backswing (≈0%–60% duration) that persisted into the forward swing (≈60%–100%). This observation was supported by the linear envelope of the EMG signals, which maintained a fluctuating but sustained amplitude across both phases. In contrast, the latissimus dorsi (Figure 6B) showed minimal recruitment during the backswing, followed by a series of high-amplitude bursts immediately after the phase transition, peaking in the mid- to late-forward swing phase. The shoulder complex (Figure 6) showed divergent recruitment patterns. The infraspinatus maintained stable, sustained activation throughout the entire stroke, with a small but statistically significant difference between phases (*p* < 0.05; d = 0.681) (Figure 6C). In context, the latissimus dorsi was specifically recruited during the forward swing, exhibiting significantly greater mean activation (*p* < 0.001; d = 1.118) (Figure 6D).

**FIGURE 7:**
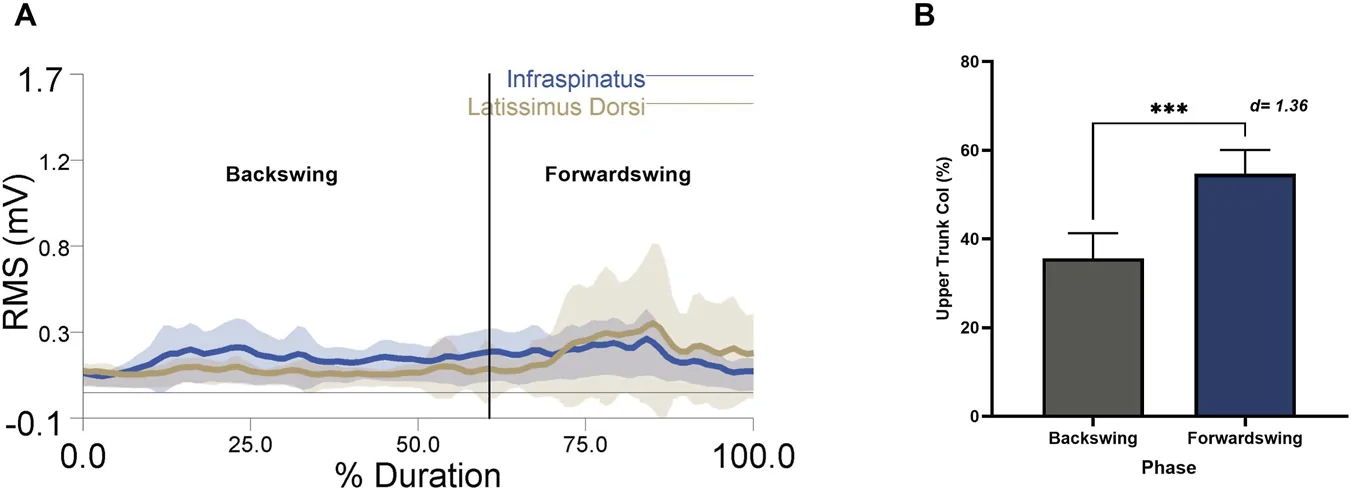
Averaged EMG linear envelope signals of the upper trunk muscles (infraspinatus and latissimus dorsi) during the para-badminton smash stroke across the backswing and forward swing phases. **(A)** Linear envelope of the EMG signal waveforms represented by solid lines, with shaded areas indicating standard deviation across all participants. **(B)** Mean upper trunk co-activation index (CoI, %) values with 95% confidence intervals during the backswing and forward swing phases across all participants. Asterisks indicate significant differences between phases (*** *p* < 0.001).

Consequently, Figure 7A shows the linear envelope of the EMG signals recorded from the selected shoulder muscles (infraspinatus and latissimus dorsi). This resulted in a significantly greater upper trunk CoI during the forward swing (*p* < 0.001; d = 1.36) (Figure 7B), reflecting a shift toward greater joint compression and stabilization during the acceleration phase.

**FIGURE 8:**
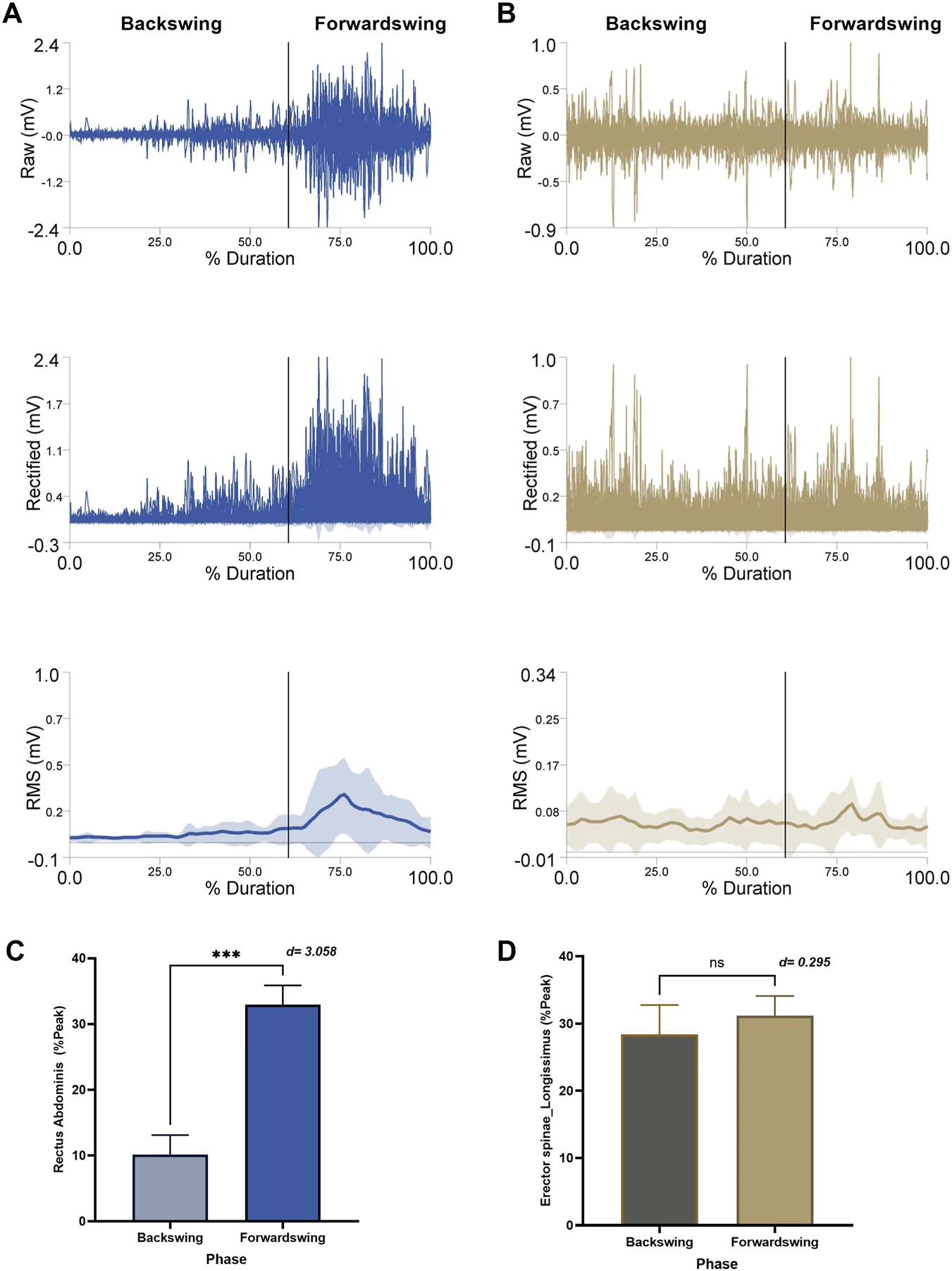
Averaged EMG activity waveforms of the lower trunk muscles during the para-badminton smash stroke across the backswing and forward swing phases, representing mean data across all participants. Rectus abdominis **(A)** (first row) raw EMG signal, (second row) rectified EMG, and (third row) linear envelope of the EMG signal. Erector spinae_longissimus **(B)** (first row) raw EMG signal, (second row) rectified EMG, and (third row) linear envelope of the EMG signal. Mean values and confidence intervals of normalized EMG amplitude (%Peak) are presented for the rectus abdominis **(C)** and erector spinae_longissimus **(D)**. Asterisks indicate significant differences between phases (*** *p* < 0.001; ns indicates non-significant).

The EMG activity of the lower trunk (part of the core musculature), comprising the rectus abdominis (RA) and erector spinae longissimus (ES), is illustrated in Figure 8. For the rectus abdominis (Figure 8A), both raw and rectified signals demonstrated low-level activity during the backswing phase, followed by a substantial increase in recruitment during the forward swing phase. The linear envelope of the EMG signals confirmed this transition, showing a prominent peak during the mid-forward swing (approximately 75% of the movement cycle). Conversely, the erector spinae longissimus (Figure 8B) exhibited a more consistent activation profile throughout the stroke. Although the signal showed intermittent bursts, the overall amplitude remained relatively stable across both the backswing and forward swing phases. Lower trunk musculature analysis (Figure 8) revealed that the erector spinae longissimus provided continuous, stable postural support across both phases, with no significant change in activation (*p* > 0.05; d = 0.295) (Figure 8D). Conversely, the rectus abdominis was dynamically recruited during the forward swing (*p* < 0.001; d = 3.058) (Figure 8C), with linear envelopes peaking at approximately 75% of the cycle.

**FIGURE 9:**
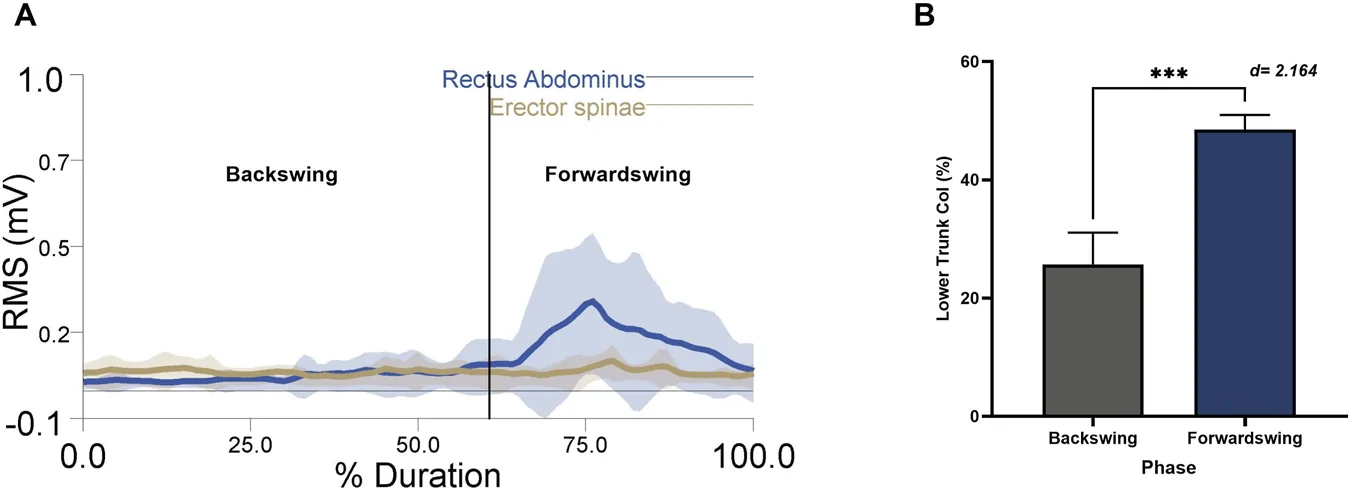
Averaged EMG linear envelope signals of the lower trunk muscles (rectus abdominis and erector spinae_longissimus) during the para-badminton smash stroke across the backswing and forward swing phases. **(A)** Linear envelope of the EMG signal waveforms represented by solid lines, with shaded areas indicating standard deviation across all participants. **(B)** Mean lower trunk co-activation index (CoI, %) values with 95% confidence intervals during the backswing and forward swing phases across all participants. Asterisks indicate significant differences between phases (*** *p* < 0.001).

Consequently, Figure 9A shows the linear envelope of the EMG signals recorded from the lower trunk musculature (rectus abdominis and erector spinae longissimus). This result led to a significantly greater elevation in the lower trunk CoI during the forward swing (*p* < 0.01; d = 2.164) (Figure 9B).

## 4 Discussion

The present study aimed to investigate muscle activation and co-activation across the backswing and forward swing phases of the para-badminton smash to improve understanding of phase-specific neuromuscular coordination. The findings demonstrate a consistent phase-dependent neuromuscular organization characterized by a transition from preparatory, low-to-moderate activation during the backswing to high-intensity, coordinated recruitment during the forward swing. This shift is accompanied by joint-specific modulation of co-activation, reflecting differing functional demands across the kinetic chain.

WH2 para-badminton athletes exhibit unique biomechanical and neuromuscular demands due to the limited contribution of the lower limbs during court movement and stroke execution. Unlike able-bodied badminton players, who rely on lower-limb force production and ground reaction forces to facilitate proximal-to-distal energy transfer, WH2 athletes depend predominantly on trunk rotation and upper-limb musculature to generate racket velocity and maintain postural stability during overhead strokes (Goosey-Tolfrey and Moss, 2005; Strapasson et al., 2021; Alberca et al., 2024). Although WH2 players generally possess greater trunk control and functional mobility than WH1 athletes, the seated playing position still constrains lower-body involvement and modifies force-transfer mechanics throughout the kinetic chain (Strapasson et al., 2021).

In addition, temporal analyses of para-badminton competition have shown that matches involving WH2 athletes are typically characterized by higher playing intensity and greater movement demands than WH1 competition (De Oliveira Mota Ribeiro and Bezerra de Almeida, 2020; Strapasson et al., 2021; Alberca et al., 2024). These increased physical and technical demands may require enhanced neuromuscular coordination and rapid upper-limb force production during stroke performance (De Oliveira Mota Ribeiro and Bezerra de Almeida, 2020; Strapasson et al., 2021; Alberca et al., 2024). Consequently, the trunk, shoulder, elbow, and wrist musculature may require elevated co-activation to increase joint stiffness, improve segmental stabilization, and optimize force transmission during high-velocity smash actions (Pincivero et al., 2003; Hortobagyi et al., 2009). However, the repetitive reliance on upper-body force generation may also increase mechanical loading and fatigue-related stress on the shoulder and wrist joints, potentially contributing to overuse injury risk in wheelchair athletes (Curtis and Black, 1999). Therefore, the joint-specific co-activation patterns observed in the present study may represent functional neuromuscular adaptations to the unique mechanical constraints and performance demands of WH2 para-badminton.

At the wrist, both the flexor carpi ulnaris and extensor carpi ulnaris exhibited significantly greater activation during the forward swing, alongside a marked increase in CoI. This pattern suggests a deliberate stiffening strategy to enhance joint stability at shuttle impact as badminton is a high-intensity sport that requires players to perform multiple movements in a short time at high speed (Masu and Nagai, 2016). Elevated antagonist co-activation at distal joints has been associated with improved joint compression and resistance to perturbation during high-velocity tasks (Kibler et al., 2006; Ellenbecker and Cools, 2010). However, excessive or repetitive co-activation may increase compressive loading across the wrist joint, potentially predisposing athletes to overuse conditions such as tendinopathy or ulnar-sided wrist pain, which are common in racket sports such as badminton, which is classified as an explosive sport (Fahlström et al., 2006; Gusliandi et al., 2020). Therefore, although co-activation appears functionally necessary for impact stabilization, its magnitude and timing should be optimized in training.

At the elbow joint, both the biceps brachii and triceps brachii demonstrated increased activation during the forward swing phase without a corresponding change in the co-activation index. These findings reflect the simultaneous activation of agonist and antagonist muscle groups surrounding the elbow joint during dynamic movement execution (Frey-Law and Avin, 2013; Murphy et al., 2022). The relatively stable co-activation pattern despite increased activation amplitudes may indicate maintenance of neuromuscular coordination and joint control during high-velocity force production. Previous biomechanical studies have suggested that balanced agonist–antagonist activation may contribute to controlled energy transfer and dynamic joint stabilization during overhead movements (Escamilla and Andrews, 2009). However, the present findings should not be interpreted as direct evidence of reduced injury risk or mechanical joint loading as EMG amplitude and co-activation indices alone cannot quantify internal tissue stress, compressive forces, or joint kinetics. Instead, the observed neuromuscular patterns may provide indirect insight into motor control strategies used to stabilize the elbow joint during the para-badminton smash. Therefore, future studies integrating synchronized EMG, kinematics, and kinetic analyses are needed to better clarify the relationship between elbow muscle coordination, mechanical loading, and potential injury mechanisms in para-badminton athletes (Segal et al., 2015).

At the shoulder, divergent muscle recruitment patterns were observed. The infraspinatus maintained consistent activation across both phases, supporting its role in dynamic glenohumeral stabilization. This aligns with its established function in maintaining humeral head centering during overhead movements (Reinold et al., 2004). Conversely, the latissimus dorsi showed phase-specific recruitment, with significant activation during the forward swing. The resulting increase in shoulder CoI suggests enhanced joint compression during acceleration, contributing to force transmission from the trunk to the upper limb. Although this coordinated activation is essential for performance, excessive reliance on powerful internal rotators such as the latissimus dorsi may contribute to muscular imbalances and increase the risk of shoulder impingement or rotator cuff overload if not counterbalanced by adequate rotator cuff conditioning (Cools et al., 2010).

The lower trunk musculature exhibited complementary activation strategies, with the erector spinae providing continuous postural support and the rectus abdominis showing phase-specific activation during the forward swing. The significantly greater trunk CoI during this phase highlights the importance of trunk stiffness in facilitating effective force transfer along the kinetic chain. This finding is consistent with the previous literature emphasizing the role of proximal stability in optimizing distal mobility and improving neuromuscular coordination in overhead sports (Kibler et al., 2006). Insufficient trunk control has been associated with compensatory loading at the shoulder and upper extremity.

Collectively, these findings support the concept of a proximal-to-distal sequencing strategy, in which force generation and stabilization are coordinated across segments to achieve efficient and safe movement execution. Importantly, the observed joint-specific differences in co-activation indicate that injury prevention programs for para-badminton athletes should not adopt a uniform approach but rather target the unique functional demands of each joint.

Although elevated muscle co-activation may enhance joint stiffness, dynamic stabilization, and movement control during high-velocity overhead actions, excessive or prolonged co-activation may also impose important physiological and mechanical costs. Increased simultaneous activation of agonist and antagonist muscles elevates compressive joint loading and muscular energy expenditure, potentially increasing mechanical stress on articular structures and periarticular tissues (Pincivero et al., 2003; Hortobagyi et al., 2009). In wheelchair para-badminton athletes, who rely predominantly on the upper limbs for both sport-specific movement and wheelchair propulsion, repetitive high-intensity co-activation at the wrist, elbow, and shoulder may accelerate neuromuscular fatigue and contribute to overuse-related conditions during prolonged match play. Excessive shoulder co-activation, for example, may increase glenohumeral joint compression and rotator cuff loading, whereas elevated wrist co-activation may increase distal joint stress during repetitive smash execution (Curtis and Black, 1999; Garcia-Vicencio et al., 2024). Therefore, the observed co-activation patterns may reflect neuromuscular coordination strategies associated with movement control during the para-badminton smash. These patterns may also be related to mechanical loading and fatigue under repetitive competitive conditions; however, such interpretations remain hypothetical because joint loading, tissue stress, and injury outcomes were not directly measured in the present study.

From a practical perspective, the present findings provide preliminary insight into the phase-specific neuromuscular coordination strategies employed by WH2 para-badminton athletes and may help inform conditioning approaches that consider the joint-specific demands of the smash stroke. The elevated wrist co-activation observed during the forward swing phase suggests that wrist stabilization and forearm neuromuscular control exercises may be important for managing impact-related loading and maintaining distal joint stability during repetitive overhead actions. In contrast, the relatively balanced agonist–antagonist activation at the elbow indicates the importance of preserving muscular symmetry and endurance around the elbow joint to maintain movement efficiency during repeated high-intensity strokes. At the shoulder, the increased activation demands placed on the latissimus dorsi and surrounding musculature highlight the need for rotator cuff endurance training, scapular stabilization, and posterior shoulder strengthening to reduce compensatory overload and maintain glenohumeral control. Additionally, the observed trunk activation patterns emphasize the importance of seated core stabilization and dynamic postural control training to improve proximal stiffness and facilitate efficient force transfer in the absence of lower-limb contribution. Given the repetitive reliance on the upper limbs for both wheelchair propulsion and stroke production, these targeted neuromuscular strategies may help reduce cumulative upper-limb loading and fatigue-related injury risk while supporting performance in WH2 para-badminton athletes.

Several limitations should be acknowledged. First, the study focused exclusively on a specific classification of para-badminton players (WH2), which may limit the generalizability of the findings to other impairment groups. Second, although EMG provides valuable insight into neuromuscular activation, it does not directly quantify joint loading or tissue stress. Accordingly, future studies are recommended to integrate synchronized EMG, kinematic, and kinetic analysis to provide a more comprehensive understanding of the relationship between muscle coordination strategies, mechanical loading, and potential injury mechanisms in para-badminton athletes. Furthermore, the relatively small sample size, inherent to research involving elite para-athletes, should be considered when interpreting the results. Future investigations with larger cohorts and multi-center collaborations are recommended to confirm and extend these findings.

## 5 Conclusion

The para-badminton smash demonstrates a phase-specific neuromuscular coordination strategy, characterized by increased joint-specific muscle activation and co-activation during the forward swing phase. The wrist exhibited elevated co-activation for impact stabilization, while the elbow maintained balanced agonist–antagonist activity, and the shoulder and trunk modulated activation patterns to support proximal stability and efficient force transfer. These findings indicate that muscle co-activation in elite WH2 para-badminton athletes is segment-specific and adapted to the mechanical demands of each joint within the kinetic chain. From an applied perspective, excessive wrist co-activation and insufficient proximal stabilization may contribute to increased upper-limb loading and potential injury risk. These findings provide preliminary insight into the joint-specific neuromuscular demands of the para-badminton smash and may inform future conditioning and rehabilitation strategies that emphasize wrist stabilization, balanced elbow coordination, rotator cuff and scapular control, and trunk stability. However, these findings should be interpreted cautiously as this study represents an exploratory, hypothesis-generating investigation based on a limited sample of elite para-badminton athletes. Future studies integrating EMG with kinematic and kinetic analysis are recommended to further investigate neuromuscular coordination and injury-related mechanisms in para-badminton.

## Data Availability

The raw data supporting the conclusions of this article will be made available by the authors, without undue reservation.
